# The Nursing Supplement

**Published:** 1889-10-05

**Authors:** 


### The Nursing Mirror.

All Contributions for this Supplement should be addressed to the Editor, THE HOSPITAL, 139-140, Salisbury Court, Fleet Street, London, E.C., and should have the word "Nursing" plainly written in left-hand top corner of the envelope.

### En Passant.

FROM FAR ISLES.—Few of us know much of the small island of St. Kilda off the coast of Scotland; and yet nurses are known and appreciated there. One of them lived at this lonely spot for long, but left last July and has not been replaced. She was kept there free of any charge to the islanders. Since she left the people have sent notice to the proprietor that "if a nurse is not sent they will all leave the island." Some time ago the proprietor offered to be at the expense of getting one of the native women instructed in the duties of a nurse, but none of them could be induced to go south for the purpose.

EXETER WORKHOUSE.—Of the state of things at present existing in Exeter Hospital under the Board of Guardians, perhaps the less said the better, only as a proof of how a nurse may be situated in such a place we quote the following: "Mrs. Babbage, nurse in the hospital ward at the workhouse, wrote saying that having been desired to tender her resignation, she should esteem it a great favour if the guardians would let her know for what reason she was called upon to resign. She added that she discharged the duties for three years and five months, and had in no way committed herself. Mr. Farmer said he did not think any of them would dismiss a servant without a fault. In this case the nurse had not been told why she was dismissed. There had been no complaint, and under the circumstances he thought the decision of the court very cruel to dismiss an officer who had done her work so well. He admitted that Mrs. Babbage had a temper, but there was an officer over her who had a temper quite as bad. Mr. Surridge: I think you had better leave her alone. Mr. Farmer: I think it is very cruel. Mr. Reed: There is no doubt she is kind and nice to the patients, but she is mischievous (laughter). Mr. Brewster, as a visitor, said that a fortnight ago he reported to the Board with reference to the bad state of the clothing in the hospital. His attention was first called to it by the matron, who fixed the charge upon the nurse. His coadjutor (Mr. Brown) took one of the stockings in his pocket and asked the nurse to explain. She said, 'How can I help it when I am supplied with nothing better. I am supplied with the same stockings week after week.' Since then he had visited the workhouse twice with Mr. Brown, and attention was called to the want of slippers in the hospital. He saw a woman shuffling about assisting the nurse in such wretched 'makeshifts of slippers and wretched stockings that were a disgrace to humanity.' The attention of the matron was called to this, and she replied that the nurse had applied for the slippers and she promised to procure them. On the following week Mr. Brown asked if they had been obtained, and the matron said they had, but she had not served them to the nurse. A few days later the matron, on being again asked, said she had not supplied the nurse with the slippers, but had sent boots instead. These boots were heavy and unfit to be worn in the hospital. The poor woman he had referred to was still shuffling about from bed to bed with the same slippers. Mr. Farmer moved as an amendment that the nurse be supplied with the reasons for her dismissal. Mr. Hamlin seconded. He added that the nurse had kept the hospital scrupulously clean, and had always done her utmost to discharge her duties properly and faithfully. The amendment was defeated by 12 votes to 17, and the original motion was carried by 13 votes to 6." Oh, for a couple of lady guardians at Exeter!

BIRKENHEAD NURSES' INSTITUTE.—A sale of work, presided over by the nurses, was held at this institute on September 26. The object of the sale was to provide a sick nurse for St. Peter's parish, and also to give the sick poor the nourishing food during the winter which is so necessary. There was a very large and fashionable attendance at the sale, and the many nurses in uniform looked very nice, and worked very hard. The lady superintendent also gave her aid.

SHORT ITEMS.—It is reported that Miss Mollett is going to South Africa to organise a nursing institution at Kimberley: we cannot but grudge her great talents to such a far-off field.—Our readers in workhouse infirmaries will regret to hear that Miss Wilson has been obliged to go abroad for three months for her health.—At Mile End Infirmary the nurses cannot be allowed to attend their respective places of worship on Sunday, as Dr. Robinson says there are not enough nurses to do the work under these circumstances.—Several nurses of Whitechapel Infirmary have resigned.—"Errors in Women's Dress" formed a subject of discussion at the British Association meeting.—The *Sunday at Home* for September contained an account of the training school for nurses connected with Marylebone Infirmary.—The private nurs ng institute connected with Bristol Infirmary will be opened early next year.—The nurses of Armagh Infirmary have been terribly overworked ever since the late railway disaster crowded their wards.

INFIRMARY NURSING.—News, both cheering and discouraging, comes from different Boards of Guardians. From Richmond we hear the following notice has been passed: "That a committee be appointed to consider the feasibility of substituting trained and paid nurses for pauper nurses throughout the workhouse." From another infirmary we hear that the guardians in selecting a matron passed over several certificated and trained nurses who presented themselves, and elected a woman without a certificate. Not long ago a widow was elected matron of an infirmary on the condition that she went to a London hospital for three months' training. No doubt the widow badly needed employment, but the benefit of the many can only be obtained in hospital work by having a trained nurse and a lady as matron. Once more we appeal to all guardians, and especially to lady guardians, to strive towards the proper nursing of every workhouse infirmary throughout the country. Paupers might at least be carefully tended at the last, when their sad lives are drawing to an end.

SALISBURY INFIRMARY WALK.—There is an old custom at Salisbury, which was carried out on September 19. A procession of patients, nurses, chaplain, and house surgeon marched to the Council House, where they were joined by the Mayor and Corporation, and then all marched to the cathedral, where a special service was held. The preacher was Canon Whiteford, and in the course of his sermon he said one noticeable point of our hospitals was the happy development by which many a woman whose life would be otherwise frivolous, listless, aimless, hopeless, was transfigured by the very act of its consecration to the sick. He knew nothing in the signs of the times better and happier than the free scope now given to that which was an instinct with all good women, sometimes with the best of them a passion—the tending of the sick. The devotion of some nurses had enriched the world, which would have been the poorer without the poetry of such a life as that of Florence Nightingale. After service the Countess of Radnor, Lady Hulse, and other ladies held the collection plates, and a sum of £46 was taken.

### Case Taking.

### SECOND PAPER.

In our first paper we considered what notes should be taken of the patient's personal history, family history, and history of present illness; this week we go on to consider the patient's condition as seen by the nurse. The general appearance and state of the patient should first be noted: if he seems weak and restless, if he lies very flat in bed, or with arms constantly raised above his head, if he seems thin, ill-nourished, or has any nervous habits, let them be written down. Then the circulation, respiration, and digestion claim attention, and with regard to these points the temperature, pulse, and respirations should be noted on a chart. A nurse should always carry a clinical thermometer and a watch with a second hand. In a large hospital it may never happen that a nurse actually requires to count either pulse or respirations, but there can be no harm in her doing so if she has time, and should she afterwards take duty in some out-of-the-way spot, she will assuredly be glad of the experience. Be it remembered we are writing for nurses who have neither students nor dressers to do the case taking.

In noting the temperature state if there is any sign of perspiration. In noting the pulse, state if it beats full or is easily compressible, or if it beats like a thin thread vibrating beneath the fingers; note the state of the veins and arteries. In noting the respiration, state if the breathing is laboured, shallow, catching, or accompanied by any peculiar noise. The expectoration must also be noticed. The significance of night perspirations, a quick thin pulse, or a crowing sound in the breathing, may or may not be known to a nurse; it is not so much her business to know as to note. She must train her powers of observation till no symptom ever escapes her quick critical eye, and then this constant observation will gradually teach its own lesson.

The state of the digestive organs can be discovered by examining the tongue, teeth, and throat, and by asking questions about any abdominal pain, or feeling of sickness. Note if the tongue is tremulous, note its colour, and if furred, or glazed and hard. Note if it is aphthous, or presents a strawberry appearance. Decayed teeth, furred teeth, or transparent-looking teeth should be noted. The state of the throat is difficult to discover merely with the spatula, which is a nurse's sole throat instrument, still, she can ask if it is sore, and see if it is swollen. Ask the patient about his appetite, and discover if there is any pain or fulness after food. Note if any vomiting has occurred.

The condition of the nervous system is shown by headpains, by movements of the eyes, the size and state of the pupils, by the sleeplessness of the patient, by his temper and spirits, and susceptibility to noise. The mental state and amount of intelligence shown should be observed, also any œdema, anæmia, orthopnœa, etc. The state of the glands of the neck, the skin, whether moist or dry, and any peculiarities of motion of the limbs should be noted, especially any local abnormalities.

The description of the actual seat of disease, its appearance, and the sensations connected therewith, must be carefully observed. In case-taking in a children's ward, ask the history of the mother's health during pregnancy, and note miscarriages. Examine the child carefully for signs of rickets or syphilis, and observe the state of the umbilicus.

The case once taken, daily notes must be kept of the patient's condition, progress, and treatment. The temperature, etc., will, of course, be noted twice daily on a chart as usual; but if the nurse observes the treatment also, she will be able to take an intelligent interest in what would otherwise be mere dry detail. In surgical cases full notes must be taken of any operation; what anæsthetic is used, how the patient bore the operation, how many sutures or drainage tubes were used, etc. When any dead bone comes away always mention the fact; and if any growth is removed by the surgeon's knife, be sure and note the size of the part taken away.

In concluding a case always try and trace a patient's convalescence to full recovery, or to give the exact cause of death. If a patient is sent away to a convalescent home, note his state after his return. If a post mortem is held on a deceased patient try and learn what was the result, and so conclude your case-taking properly.

(*To be continued.*)

### Answers to Examination Questions.

By a Country Workhouse Nurse.

Question I.—To what special points should you pay attention in nursing a case of typhoid fever, scarlet fever, rheumatic fever?

*Typhoid Fever.*—1. To the spots; their number and appearance; to any distension of the abdomen.

2. To the motions; their number, character, colour must be noted, and the least trace of hœmorrhage reported to the doctor.

3. To the cough; its frequency, the amount of expectoration, and nature, any undue quickening of the breathing to be reported at once, before it approaches distress.

4. Any symptoms of pain or tenderness in a fixed spot over abdomen to be reported at once, rigor noticed, and reported.

5. The efforts of the nurse must be directed to saving the patient from every exertion; to keeping up his strength by the utmost regularity in the giving of food, etc., and to never letting him sit up, either to feed him, or for examination.

6. Temperature usually every four hours; pulse and respiration at least twice a day, as the doctor orders.

7. From the beginning of the illness precautions against bed sores must be taken, by washing the patient's back with soap and water, and rubbing it with methylated spirits of wine. Of course, should the sheets be soiled, care must be taken to change them at once. It is well to wash out his mouth and lips twice or three times a day with glycerine and borax. This will probably prevent him or her from suffering from thrush, or cracked, hard lips, things which feverish patients are apt to have, and which cause much unnecessary annoyance.

8. Careful disinfection of linen, bed pans, etc., as the least carelessness may carry the disease to others. Soiled linen should be removed at once, soaked in carbolic acid, 1 in 20 solution, or perchloride of mercury, solution 1 in 100, and then boiled; bed pans, etc., plunged in very hot water, and then washed out in 1 in 20 carbolic. The nurse should be careful to wash and carbolise her hands each time she attends to the patient, and as a routine, she should wash and carbolise her hand always before eating. All washing of patient to be done with tow or absorbent wool, which must be burnt. It is well to hang a sheet soaked in carbolic, or a large towel, on a clothes-horse, which seems to freshen and purify the air of the room, and saves the nurse much unpleasantness. A window should always be open.

*Scarlet Fever.*—1. Carefully watch urine, especially from middle of second week; test it daily for albumen (if the doctor wishes it—some doctors do not like the nurse to test). Report at once if it lessens in quantity, looks smoky, or is more frequent than usual. It is well to keep a specimen passed during the night for the doctor to see in the morning, and another passed during the day for his evening inspection.

2. Report immediately any puffy look about the patient's eyelids in the morning, any signs of pitting on pressure about feet and ankles; all which symptoms may point to kidney trouble.

3. Watch for discharge from ear, swelling of glands of neck, or of joints.

4. Disinfect all clothing by plunging it in a disinfecting solution, and by boiling; disinfect cups, feeders, etc., and all things used by patient; also your own clothes. The peeling time is the most dangerous time for propagating the infection; the patient should be oiled all over twice a day, and have hot baths as soon as allowed. These precautions prevent the particles of skin from flying about, and carrying the illness to others.

*Rheumatic Fever.*—1. Put the patient, if possible, on a water-bed from beginning of attack, and between blankets. The water-bed will save him much suffering, as it will be so much easier to prevent bed sores, and to move him without causing pain.

2. As the heart is liable to be affected, it is important not to allow the patient to sit up for any purpose whatever; and to prevent his making any sudden movements or efforts.

3. Give the doctor an idea of the quantity and quality of the perspiration.

4. Take great care of the positions of limbs, so that they don't get stiff or bent, move joints gently about as soon as patient can bear it. Massage during convalescence is useful. Take temperature, pulse, and respiration, as the doctor orders, usually every four hours. Keep the room warm, but fresh.

### An Exhibition of Nursing Uniforms.

### THE DOLL COMPETITION.

We wish to call the attention of all matrons to the following list of hospitals which will be represented at the forthcoming doll show, and to point out that our object is to establish a useful and permanent reference regarding nursing uniforms. Matrons and nurses will then be able to choose or recognise any uniform they wish or see. To make the exhibition complete, we would ask those matrons whose hospitals are not mentioned below to bring the matter before their nurses. Dolls will be received at The Lodge, Porchester-square, London, W., between Nov. 24th and 31st, but not *before* the first date. The Editor has been considerably embarrassed already by the arrival of some very charming dolls, which, nevertheless, look somewhat out of place in his study. A committee of three, consisting of two matrons of hospitals and the Editor, will award the £10 in prizes as they shall see fit. We regret that last week an incomplete list of institutions competing was given, and that the last day of entrance was given as October 1st, instead of *October 31st*. Names will gladly be received till the latter date.

Birmingham Institution.
Brompton Cancer Hospital.
Bucks. Infirmary.
Cheltenham Nursing Institution.
Cork Children's Hospital.
Crumpsall Infirmary.
Deaconesses' Institution, Tottenham.
Devon and Exeter Hospital.
Derby Children's Hospital.
Edinburgh Royal Infirmary.
Fir's Home, Bournemouth.
Glasgow Sick Poor Association.
Glasgow Western Infirmary.
Glasgow Hospital for Children.
Great Northern Central Hospital.
Grimsby and District Hospital.
Home Hospital, Weymouth-street.
Home Hospital, Fitzroy House.
Home Hospital, Hampstead.
Hahnemann Hospital, Liverpool.
King's College Hospital.
London Hospital.
London Fever Hospital.
Leeds Nurses' Institution.
Leicester Infirmary.
National Hospital, Queen's-square.
Northampton Nursing Institution.
Northwich Victoria Infirmary.
Norwich Nurses' Home.
Pendlebury Children's Hospital.
Private Home, Portland-place.
Radcliffe Infirmary.
Royal Albert Hospital, Devonport.
Royal Berks. Hospital.
Sheffield Children's Hospital.
South Devon Hospital.
Stamford Nursing Society.
St. Helen's Institute, Jersey.
St. John the Evangelist Institute, Strand.
St. Mary's Hospital, Paddington.
St. Marylebone Infirmary.
St. Thomas's Hospital.
Sussex County Hospital.
Truro Infirmary.
University Hospital, London.
Wandsworth and Clapham Infirmary.
Warneford Hospital.
West Malling Private Institute.
Wilkington Hospital, Manchester.
Winchester County Hospital.

### Presentations.

Last week at St. George's-in-the-East Infirmary a very handsome piece of plate was presented by the nurses to Miss Hughes, matron, who is leaving, having recently been appointed matron to the Kensington Infirmary. It bore the following inscription: "Presented to Miss F. M. Hughes, matron of St. George's Infirmary, by the female staff, on her leaving, as a slight token of their respect and affection. September 19, 1889." This was accompanied by a note: "We, the undersigned, ask you to accept this small gift as a token of our sincere regard and gratitude for all the kindness shown during the period of your matronship among us, wishing you every success in the future." Then followed the signatures of twenty-nine members of the staff.

Miss Thomas, secretary to the ladies' classes of the St. John's Ambulance Association at Buxton, has been presented with a silver medallion in recognition of her services.

Sister Maria Theresa, Superior of the Sisters of Mercy in Tong-king, has just been presented with the Cross of the Legion of Honour. For over thirty-one years she has ministered to the wounded of the French army, and has herself been wounded on three occasions—at Balaclava, Magenta, and Wörth. During the war with Germany she once courageously seized a smoking shell, which had fallen in the midst of the field hospital, and ran with it into an adjoining meadow, where she was severely injured by its explosion. In Syria, China, Mexico, and now in Tong-king, Sister Theresa has followed the French armies with the most extraordinary intrepidity and devotion. Wherever the enemy's fire was hottest there might Sister Theresa be seen, binding up wounds, or soothing last moments. It is not surprising to learn that she was received with immense enthusiasm when the cross was presented to her the other day by the French Governor of Tong-king, in presence of all the troops of the garrison.

### Lectures.

Dr. Kate Mitchell will lecture on Physiology and Health at 90, Upper Tollington-street, on October 31 and November 7 and 14. Fee for the course, 4s. 6d. Dr. Kate Mitchell will also lecture at Colchester in October and at Bournemouth in November, the latter course being for ladies only.

Mr. E. A. Parkyn is delivering a course of lectures at Gresham College, E.C., on Mondays, at 7.30. Subject, "The Nervous System. Fee for the course, 5s.

Mr. E. A. Parkyn will commence a series of lectures on Human Physiology at Hornsey National Hall, commencing October 11. Fee, 5s.

Dr. Sanways will deliver a course of lectures at St. John's Schoolroom, New Southgate, N., on the Body and Health, commencing October 7, at 8 p.m. Fee, 5s.

Mr. Walter Pye will deliver a series of lectures at Toynbee Hall on Personal and Social Hygiene, commencing October 4, at 8 p.m. Fee, 1s.

### Appointments.

[It is requested that successful candidates will send a copy of their applications and testimonials, with date of election, to The Editor, The Lodge, Porchester-square, W.]

St. George's-in-the-East Infirmary.—The guardians have appointed Miss Wesley matron of this infirmary. We believe Miss Wesley has had six months training as a nurse, but her latest post has been at Banstead Schools. Coming after such a thoroughly trained nurse as Miss Hughes, who has worked the infirmary into excellent order, Miss Wesley is likely to find her position difficult.

Salford Royal Hospital.—Mr. John Farrington, M.R.C.S., L.R.C.P., has been appointed house surgeon to the Pendleton Branch Dispensary.

### Vacancies.

The following vacancies are announced

**Lady Superintendent.**

Birmingham Homœopathic Hospital.

**Matrons.**

Hayes Cottage Hospital, £25.

Hospital for Epilepsy, Regent's Park, £50.

Salisbury Infirmary, £75.

St. Leonard's Convalescent Home, £50.

York Lunatic Asylum, £60.

**Nurses.**

Arbroath Infirmary, £20.

Brecon Infirmary, £18.

Bridgwater Infirmary.

Brixton Institute.

Bury Hospital for Incurables.

Carmarthen Infirmary, £23.

Chalfont St. Peter's Hospital, £25.

Cheltenham Institution.

Chester Deaconess' Institute, £22.

Highbury Institute, £20.

Hospital for Diseases of the Chest, E., £20.

Jersey Nurses' Institute, £25.

Kent Nursing Institution (several).

Manchester Southern Hospital, £25.

Morley's Convalescent Hospital, £22

Newcastle-on-Tyne Nurses' Home (six).

North Devon Infirmary, £20.

North-Western Fever Hospital, £30.

Nurses' Home, Hull, £25.

Nursing Institution, 96, 97, 98, Wimpole-street, London, W., Mr. Wilson has several vacancies for thoroughly trained nurses.

Park-lane, Croydon, £22.

Scarborough Institute.

Sheffield Nurses' Home.

South-Eastern Fever Hospital, £30.

Southport Nurses' Home (several), £20.

St. Helena Home, N.W. (several),

St. John's Institute, Southport.

Victoria Home, Bournemouth, £25

Western Fever Hospital, £22.

West Bromwich District Hospital (two), £22.

Weston-super-Mare Hospital (night)

York Nurses' Institution.

**Midwife.**

Western General Dispensary.

**District Nurses.**

Bedfordshire Nurses' Institute, £26

Blackheath Institute for Trained Nurses, £25.

Crediton, Devon, £60.

Nurses' Home, Frome, £20.

**Probationers.**

Birmingham Eye Hospital.
Grimsby Hospital.
H. M. Hospital, Stepney (several).
Horton Infirmary.
Jarrow-on-Tyne Memorial Hospital.
Taunton Hospital.

### Notes and Queries.

To Correspondents.—1. Questions may be written on post-cards. 2. Advertisements in disguise are inadmissible. 3. In answering a query please quote the number. 4. If a private answer is desired, a stamped addressed envelope must be forwarded.

Notice.—We must really ask our correspondents to be more particular in enclosing name and address. Constantly we receive queries or letters with no name attached.

### Queries.

*Home for Chronic and Incurable Diseases.*—"Kappa" writes to ask if there is any institution for chronic and incurable cases where he can find a home. He is still a young man, but suffers severely from varicose veins, not only in his legs and thighs, but gradually extending over his whole body, and he is obliged to be continually in a recumbent position. Although he does not really require medical aid, his case is quite hopeless, and he is anxious to escape from the monotony and loneliness of life in private lodgings.

Can anyone suggest a remedy to get rid of stoutness in a young man, aged thirty-four? He has tried less diet, but that does not do, as with less food he feels less inclined for walking and not as lively and cheerful. *A. B.*

Inquirer would be glad of any information about the registration of midwives. *A Certificated Midwife.*

### Answers.

*Nurse J.*—The "Recollections of a Nurse" are published by Macmillan. Price 2s.

*M. B.*—The latest work on "Epilepsy" is that of Dr. William Alexander, published by Young J. Pentland. If you write to Kempton's Medical Book Stores, 4, Hanway-street, Oxford-street, they will doubtless supply you with something cheaper, second-hand.

*Sister Eva.*—A "Medical Vocabulary," by Mayne. Published by Churchill. Price 10s. 6d.

### Everybody's Opinion.

*Correspondence on all subjects is invited, but we cannot in any way be responsible for the opinions expressed by our correspondents. No communications can be entertained if the name and address of the correspondent is not given, or unless one side of the paper only be written on.*]

### EXAMINATION QUESTION.

A Would-be-Nurse writes: An examination question competition would be a great help if the best set of answers could be published. I have tried to answer all the questions published in The Hospital for the last year and a half, but cannot tell if my answers are right or not. Reading up the subjects and writing out the answers has taught me much.

### Amusements and Relaxation.

### SPECIAL NOTICE.

**ACROSTIC COMPETITION.**

**The Prize Editor having been requested by several correspondents to continue the ORIGINAL and SET Acrostic Competitions will be glad to receive the names of all intending competitors on or before October 5th.**

### .B. Third Quarterly Word Competition Commences October 5th, 1889, ends December 28th, 1889.

Three Prizes of 15s., 10s., 5s., will be given for the largest number of words derived from the words set for dissection.

Proper names, abbreviations, foreign words, words of less than four letters, and repetitions are barred; plurals, and past and present participles of verbs, are allowed. Nuttall's *Standard* dictionary only to be used.

N.B.—Word dissections must be sent in WEEKLY, arranged alphabetically, with correct total affixed.

The word for dissection for this, the *First* week of the quarter, being

"PEACOCK."

**Results of Twelfth Week.**

| Names. | Sept. 28th. | Totals. | Names. | Sept. 28th. | Totals. |
|---|---|---|---|---|---|
| Nurse Duty | 30 | 1011 | F. C. J. | — | 305 |
| Jumps | — | 30 | Percy Verance | — | 18 |
| N'importe Qui | 20 | 798 | Gleniffer | — | 175 |
| Tinie | — | 186 | Gypsye | 31 | 909 |
| Amicus | — | 430 | Ladyr | — | 127 |
| Patience | 29 | 1020 | A. B. | — | 170 |
| Broxbourne | — | 398 | Essie | — | 393 |
| Speedwell | 30 | 894 | Cowboy Bill | — | 179 |
| Lightowlers | — | 356 | M. L. A. | — | 117 |
| Lauriston | — | 345 | W. G. R. | 19 | 778 |
| Sister | 31 | 924 | Little Tuppy | — | 259 |
| Rattler | — | 28 | Embryo | — | 56 |
| Paignton | — | 334 | Jenny Wren | 25 | 751 |
| Fassifern | — | 141 | Pearl | — | 132 |
| W. R. E. | 30 | 990 | Condorcet | — | 450 |
| Buxton | — | 167 | R. W. | — | 43 |
| Nux Vom | — | 263 | Electrician | 29 | 761 |
| Esperance | 29 | 995 | Nurse Amie | — | 89 |
| Spes | — | 24 | Sunshine | 29 | 659 |
| Secnarf | — | 229 | Juvenis | — | 23 |
| M. W. | 29 | 995 | Qu'appelle | 29 | 800 |
| Rover | — | 226 | K. Jarman | — | 43 |
| Leirion | — | 195 | Reveal | — | 196 |
| Matron | — | 174 | Amos | — | 224 |
| Isabel | 28 | 550 | H.C. | 24 | 24 |

The Result of the Second Quarterly Word Competition will be published October 12th.

### Notice to Correspondents.

N.B.—All letters referring to this page which do not arrive at 139 and 140, Salisbury-court, London, E.C., by the *first post on Thursdays*, and are not addressed PRIZE EDITOR, will in future be disqualified and disregarded

### Conditions.

I. Every paper sent in must be clearly written, and one side only must be used. All competitors must mark at the head of their papers the number of their competition.

II. Each paper must be signed by the author with his or her real name and address. A *nom de plume* may be added if the writer does not desire to be referred to by us by his real name. In the case of all prize-winners, however, the real name and address will be published.

III. All communications relating to prize competitions should be addressed to "The Prize Editor, The Hospital, 139 and 140, Salisbury-court, London, E.C." Competitors are requested not to include in letters, etc., to the Prize Editor communications on matters of other business. All letters must be fully prepaid.

IV. The Prize Editor of The Hospital is the sole judge of the competitions, and his decision is in all cases final and without appeal.

V. The Prize Editor reserves the right to publish any paper sent in, whether it receives a prize or not. He does not hold himself responsible for any MSS. sent, nor can he undertake to return rejected competitions.

### Miss Valeria.

In a pleasant little house, situated in the outskirts of the classic town of Cambridge, dwelt Mrs. Forbes and her only daughter, Dorothy, a graduate of Girton College (she was bracketted twenty-fifth in classics, Class II.), and hence christened by myself, to myself, "Girtonina." Girtonina was artistic, musical, full of talent, and possessed with an insatiable desire to be the bluest of blue-stockings. Against this, however, her appearance militated strongly. Nothing could be less stately than her slight and graceful figure; nothing less forbidding than her mobile face, full of life and colour, and crowned with a coronal of blue-black hair, brushed back from a forehead somewhat too lofty to be consistent with the standard lines of perfect beauty.

Mrs. Forbes always gave us undergraduates a warm welcome, and one that put the most nervous of us at his ease; and it was much that my companions and I owed to the refining influences which the *entrée* to this pleasant home-circle subjected us.

We were a privileged few, mostly from different colleges. There was Jones, of Catherine's, something of an artist, who intended to forsake the cloister for the studio and immortalise his name on the walls of Academy or Salon; pale, gentle little Wilson, of Peterhouse, who was reading for orders; blustering Belton, of King's, who was destined for the bar; and my chum, Becher, of Trinity, philosopher, sceptic, and "old brick" in one. All these, with myself (who, possessed of a small income, had no definite aims for a profession) were frequently to be found at Mrs. Forbes's "at homes"—not one of which would we willingly have neglected to attend.

We were gathered together, on the evening I am describing, with two other members of our select circle, who deserve a word of introduction. The one, Father Wright, or "Father" as he loved to be called and we loved to call him, was a Catholic priest of a genial and kind-hearted type not uncommon in English country towns. The other was Miss Valeria. She possessed a surname, but no one ever called her by it. It was "Miss Valeria" always, and she was on capital terms with us all, being by no means the least attractive of the many attractions which brought us to Mrs. Forbes's house. She was a woman of many foibles, and, on questions affecting the position of women in the world, held "advanced opinions." She was undoubtedly the centre of our small community. Her attraction lay not so much in her rare beauty as in the romance of her life, and in the intensely earnest and unconventional cast of her mind. Her face arrested you at the first glance: a complete oval, with a complexion pale almost to pallor and a lofty intellectual forehead crowned with hair of the deepest auburn. A face one would have called cold and passionless, save for the intensity of her clear grey eyes which were deep-set and resolute. A straight elastic figure of commanding height lent her that external queenliness of aspect to which she mentally aspired, as if expressing the aspiration of her life, which was animated by a desire not to rule but to lead. Her intellect was of a high cast, but she had not at this time freed her mind from the influence of prophets.

Born in Italy, at the time of its internal revolutions, of an English father and Italian mother, Miss Valeria's girlhood had been passed in an atmosphere that echoed to the names and was saturated with the stirring ideals of Mazzini and Garibaldi. This fact explains much in her conduct which was at the time inexplicable to us, and which we mistook for eccentricity. From her earliest years she had imbibed that patriotic spirit which is so eager to seize upon the many features of the social life around it which appear to need reform. Bred in the midst of these influences, her youth freed from the restrictions, social and educational, common to the English girl, she had arrived in England impregnated with a strange enthusiasm for reform, and an intense hatred of convention very strongly felt, but to which she was unable to give adequate expression. She had come to Cambridge on account of the many opportunities for real study which the place afforded, and she spent her time mostly in a congenial solitude, feeding upon her visionary schemes.

Her precise aim she herself could hardly define, and it was scarcely her fault that she had overlooked some facts of human nature, the relation of the brain to the heart (in the homely sense of the word), and the like. She only knew that she felt an unconquerable antagonism towards the conventions of the world which, she contended, prevented that complete development of feminine faculties to which woman's equality of education with that of man entitled her. As history and the personal experience of her early life had taught her that nothing noble or great could be accomplished except through suffering, so should the victory demand a victim ere it could be won, this modern Iphigeneia was ready, metaphorically, to bare her throat to the sacrificial knife.

So, with good old Father Wright to direct us, homely Mrs. Forbes, her clever daughter, and fervid Miss Valeria to stimulate us, we whiled many a pleasant hour away at these gatherings in friendly chats on social problems, history, art, religion, and music. Then Girtonina would produce her violin, an instrument over which she had complete mastery, and move us with its almost human tones. I aspired, too, to be something more than a versifier, and I have still a guilty knowledge of the passionate rhymes I wrote under the inspiration of that instrument.

It was under such circumstances as these that I first met Miss Valeria. We enjoyed many a similar evening, until the close of the Easter term arrived, and, as many of us were quiting the university, it was not without regret that our small company regarded its approaching dissolution. Girtonina was going to London as an art-student. Miss Valeria had those tangible schemes of her own, for the fulfilment of which London, too, could only offer scope.

We spent our last evening together in having a quiet row upon the sluggish Cam. Becher and I pulled lazily over the pleasant reach at the backs of the colleges: Girtonina reclined upon a cushion in her favourite place in the bow, while Mrs. Forbes and Miss Valeria sat in the stern. We paused a moment beneath the Bridge of Sighs.

"The name is appropriate to day," said Girtonina, as she indolently fretted the water with her fingers, and playfully flicked a few drops over me.

We glide along as lazily as the sluggish stream that bears us, each occupied with his own thoughts and enjoying the calm of the evening. As the twilight gathers, half turning on my seat, I watch the shadows come and go on Girtonina's face, until something in my gaze, as it meets hers, forces a momentary blush across her features. Was it my fancy, or only the reflection of the red flush in the evening sky? Joyfully, I note her lowering eyelids and quivering lips as, unchecked, my hand clasps hers in silence.

Three years have passed away since that eventful evening, and Miss Valeria is sitting in her dull lodging situated in a street off one of those dusty squares which stud the region of Bloomsbury. It is Christmas time. Peace and good-will, those apt attributes of the season, seem to have thoroughly infected all the passers-by, but neither the subdued murmur of the parents nor the free, unrestrained laughter of the children, as they pass her window, finds its echo in Miss Valeria's heart. The sounds jar on her somewhat, but not disagreeably, only as if the chords of her heart were out of tune and failed to quiver with a responsive echo. Not that she is without sympathies, but her habits of thought have stifled for a time all those rare tender impulses in a woman's heart. They are not dead, they only sleep; but they will need a rude awakening. Her attitude as she gazes from her window at the small patch of sky murkily visible above the opposite house-tops, is one of dejection and disappointment. She was weary and dispirited, for those vague and visionary plans of action with which she had left Cambridge were no nearer realisation than before. She was a clever woman, therefore men had fought shy of her. But the innate feminine weakness which she had deluded herself into believing non-existent, was already beginning to make itself felt in the longing for a sympathiser with her wild Utopian schemes. Half unconsciously, she often murmured to herself:

"How sweet did any heart now share in my emotion!"

Since our Cambridge days the members of the little company which had met together in Mrs. Forbes's house had, more or less, lost sight of each other, and Miss Valeria remained only a recollection to them. The solitary exception was Becher. His own strong originality had drawn him towards Miss Valeria in the past, and he contrived to find many opportunities of meeting her in London.

"I am thinking of a new scheme," she said to him one day. "I am so tired of inaction. I fancy something lasting might be done if I could get a little society together, so I have resolved to hire a room and give a lecture, in the hope that a few serious-minded persons may be induced to rally round me. What do you think of it?"

"I can only admire the tenacity with which you cling to your idea," Becher answered; "but have you considered this last plan in all its aspects? Think of its boldness, and that you are in a country where to disregard convention is moral death, and puts one outside the pale of respectability."

"I hate that word 'respectability,'" she answered hastily and with warmth. "It is the cloak for all the meannesses and all the incompetencies in the world. Put only a mediocre, respectable man in a position of power and responsibility—a man without genius or originality—and he is sure to be the cause of a multitude of disturbances. Respectability is the fungus of civilisation."

"I will not deny that you are right," he said "warily" in response to this vehement outburst, "but men of genius are few, and one must work with the material that is to hand."

"Then we must find women of genius to supply the place of them," she said. "I am not to be deterred. May I count upon your help? It is a shame to ask you, though, but I have so few friends, and—sometimes I think my resolution is beginning to fail me."

There was a slight tremor in her voice, which thrilled him as she finished.

"Count on me always," he replied firmly and quickly, without giving a thought to the probable consequences; and the deep tones of his voice, no less than the steady glance from his eyes, told it was no half-hearted assistance he was offering.

"When will it take place?" he asked.

"This day week."

"What shall we call it? 'Miss Valeria's Christmas Treat?'" he asked, laughingly, as he bade her good-bye.

The cherished dreams of a life which has not yet been disillusionised are not to be dispelled by lukewarm criticism. So it came to pass that Miss Valeria hired a small hall in the neighbourhood of Islington. It was one of that class of rooms which are let out one night for a tradesman's ball, the next for the performances of a troupe of local amateur actors, the next for the meetings of a Socialist club; in short, for numerous popular *réunions*—political, social, and religious. It was also the Sunday conventicle of a smug-faced gentleman calling himself an "atheist," who delivered fervid discourses, weekly, in praise of Bentham and Rousseau, interspersing his remarks with coarse witticisms at the expense of Moses.

Miss Valeria had superintended the arrangements herself, with the assistance of the hallkeeper, and handbills had been distributed in the neighbourhood announcing that on a certain evening, at Rosslyn Hall, Derby-street, Islington, a lady would give an address on

"The Social Status of Women."

All persons interested in the subject, without distinction of sex, were invited to attend.

Becher's feelings, when he received the ominous handbill one morning, were of a mixed kind. He had an Englishman's horror of notoriety, especially female notoriety; but he remembered the thorough sincerity of Miss Valeria's enthusiasm, and also how attractive her presence was to himself. He felt a thrill of pleasure, too, at the thought that she had come to him for help.

It was whispered abroad that an eccentric lady was about to lecture publicly. No one in Miss Valeria's immediate circle had openly announced their wish to attend. A hall in some shabby Islington den! It was too outrageous. If it had been the Royal Society, now, with Professor Dryer-than-dust in the chair, it would be an altogether different thing!

When the eventful evening arrived, Becher found it necessary to brace himself together to face the inevitable. He had promised to escort Miss Valeria to the hall. Light flakes of snow fell as they passed along the streets; Miss Valeria busy with her own thoughts, and Becher too bewildered for once, to be able to rally his spirits. At length they arrived at Rosslyn Hall.

"It does not look very encouraging," she said, taking in at a glance the prevalent dinginess and tawdry decorations of the hall.

"Not very," he answered.

"But I cannot give in now," she added.

The audience was already beginning to assemble.

Miss Valeria seated herself by Becher in the front row, being unwilling to face her audience until it became absolutely necessary. The buzz of conversation grew louder as the room filled, and at last the bell of a neighbouring clock struck the hour. Slowly, but without hesitation, Miss Valeria mounted the platform.

Gradually the applause which met her died away, and she lifted her eyes and faced them. How beautiful she looked, thought Becher, and how self-reliant! Her face was calm and quiet, her figure firm and unshrinking. An involuntary tremor played for an instant round her lips; a slight heightening of the colour in her cheeks alone betrayed consciousness of her position. But all nervousness vanished at the first sound of her voice, which never for a moment faltered, as in clear ringing tones she spoke to them.

A dim recollection only of what she said remained to Becher afterwards. He knew she spoke of female labour, of girls who were drags at home. She formulated some scheme which should make them independent, and finally concluded with an invitation to any who wished to talk the matter over with her to stay behind. She poured out the pent-up passion of her life with a simple unstudied eloquence, so attractive from its earnestness, that the applause was loud and long when she closed.

Then the audience rose to go. They had had their entertainment and were satisfied. Few took any note of the subject matter, though the subtle power of her oratory, and the fascination of her voice, had arrested and stirred them vaguely.

The hall emptied completely, and, save one old man, no one ventured to stay behind. He approached Becher and wished him, in a quavering voice, the compliments of the season, remarking at the same time that the night was cold and it was a fit season to "drink his honour's health." When Becher had got rid of him, Miss Valeria descended the platform and sank, utterly wearied and dejected, upon the nearest bench.

"Are you convinced now," Becher asked, "of the futility of your method? The age of miracles is over, and you only waste your words."

"I fear you are right," she answered, and her lofty self-reliance seemed to forsake her as two tears stole silently down her cheeks.

Becher was disarmed at the sight of her grief, and he drew closer to her side. Something gave him courage to speak all that had been in his mind so often.

"Did you never think," he asked, speaking rapidly, "that your schemes are a war against Nature; that it is the function of everything feminine to reflect a ray of the masculine? Your self-reliance breaks down at a critical moment and leaves you helpless. Let me share your enthusiasm. Give me the right to support it. Here, rest here, sheltered by my arm; and if the world need reform let us reform it together."

More rapid words were spoken; some hasty, broken sentences, in reply, telling over again that old story—so old that some think the sensible world should forget it now—and Becher rose and cried with his old manner:

"Now I can live. Ah! we conjugate that verb wrongly, for its perfect tense is *love*."

"A pretty but a borrowed simile," answered Miss Valeria, with her rare smile and a dash of her old critical spirit. And, with an action so proudly confident, she twined her arm in his, as if she at last acknowledged the necessity for some support. Love is lord of all still!

Two figures lingered in the dismal little hall long after the audience had gone, the one in the conscious pride of victory, the other in mute surrender to a stronger power. The one thing wanting Miss Valeria had found at last—the guiding hand, the steadfast, trusty heart, firm and strong, but gentle, too.

Our little Cambridge circle has slipped widely apart. Some day I hope the world will hear of its individual members. Father Wright and Mrs. Forbes are still esteemed by another generation of undergraduates. And Girtonina—well, there is a figure bending over me at this time as I write, that suggests the capricious little bluestocking. A rippling laugh, which has a familiar sound, floats through the room as she glances over my shoulder. Sometimes the tones of a violin struck by a master hand echo through the passages, and these tones have less of passion than of settled peace in their cadences now.

Cuthbert Withers.

